# Controlling absence seizures from the cerebellar nuclei via activation of the G_q_ signaling pathway

**DOI:** 10.1007/s00018-022-04221-5

**Published:** 2022-03-19

**Authors:** Jan Claudius Schwitalla, Johanna Pakusch, Brix Mücher, Alexander Brückner, Dominic Alexej Depke, Thomas Fenzl, Chris I. De Zeeuw, Lieke Kros, Freek E. Hoebeek, Melanie D. Mark

**Affiliations:** 1 Department of Behavioral Neuroscience, Ruhr-University Bochum, 44801 Bochum, Germany; 2 Department of Zoology and Neurobiology, Ruhr-University Bochum, 44801 Bochum, Germany; 3 Institute of Physiology I, Medical Faculty, University of Bonn, 53127 Bonn, Germany; 4 European Institute of Molecular Imaging, University of Münster, 48149 Münster, Germany; 5 Department of Anesthesiology and Intensive Care, TUM School of Medicine, Technical University of Munich, 81675 Munich, Germany; 6 Department of Neuroscience, Erasmus MC, 3015 AA Rotterdam, The Netherlands; 7 Netherlands Institute for Neuroscience, Royal Dutch Academy for Arts and Sciences, 1105 BA Amsterdam, The Netherlands; 8 Department for Developmental Origins of Disease, Wilhelmina Children’s Hospital and Brain Center, University Medical Center Utrecht, 3584 EA Utrecht, The Netherlands

**Keywords:** Absence epilepsy, Optogenetic stimulation, Chemogenetic stimulation, GPCR, mGluR1

## Abstract

Absence seizures (ASs) are characterized by pathological electrographic oscillations in the cerebral cortex and thalamus, which are called spike-and-wave discharges (SWDs). Subcortical structures, such as the cerebellum, may well contribute to the emergence of ASs, but the cellular and molecular underpinnings remain poorly understood. Here we show that the genetic ablation of P/Q-type calcium channels in cerebellar granule cells (quirky) or Purkinje cells (purky) leads to recurrent SWDs with the purky model showing the more severe phenotype. The quirky mouse model showed irregular action potential firing of their cerebellar nuclei (CN) neurons as well as rhythmic firing during the wave of their SWDs. The purky model also showed irregular CN firing, in addition to a reduced firing rate and rhythmicity during the spike of the SWDs. In both models, the incidence of SWDs could be decreased by increasing CN activity via activation of the G_q_-coupled designer receptor exclusively activated by designer drugs (DREADDs) or via that of the G_q_-coupled metabotropic glutamate receptor 1. In contrast, the incidence of SWDs was increased by decreasing CN activity via activation of the inhibitory G_i/o_-coupled DREADD. Finally, disrupting CN rhythmic firing with a closed-loop channelrhodopsin-2 stimulation protocol confirmed that ongoing SWDs can be ceased by activating CN neurons. Together, our data highlight that P/Q-type calcium channels in cerebellar granule cells and Purkinje cells can be relevant for epileptogenesis, that G_q_-coupled activation of CN neurons can exert anti-epileptic effects and that precisely timed activation of the CN can be used to stop ongoing SWDs.

**Supplementary Information:**

The online version contains supplementary material available at 10.1007/s00018-022-04221-5.

## Introduction

Absence seizures (ASs) with their characteristic bilateral thalamic and cortical electrographic manifestations called spike-and-wave discharges (SWDs) are the hallmark symptom of various idiopathic generalized epilepsies [1, 2]. During these recurrent non-convulsive 2.5–4 Hz pathological oscillations with sudden onset and termination, patients suffer from impaired consciousness and automatisms such as twitches [3]. Although rodent models of ASs differ in SWD frequency from human patients (rats 7–11 Hz and mice 5–7 Hz), they mimic the human disease characteristics in that they include behavioral arrests and orofacial twitches during SWDs, as well as the unique pharmacological sensitivity to the anti-epileptic drug, ethosuximide (ETX) [4, 5]. Different studies have shown that the rhythmic cortical activity seen during SWDs originates from reciprocal interplay between neocortical pyramidal cells, thalamic cells and neurons of the thalamic reticular nucleus [6–12]. During SWDs, these neocortical and thalamic neurons fire action potentials rhythmically during the spike of the SWDs, whereas they pause during the wave, together providing the cellular basis for the waxing and waning pattern of the electrographic spindles [13–16]. In addition to the thalamus and cortex, the basal ganglia and cerebellum also show modulations of action potential firing during SWDs [17–19]. Importantly, abnormal activity in the cerebellum can probably not only be the consequence of epileptic activity in the brain, but also the cause thereof. Indeed restricted knockouts of the P/Q-type calcium channel in cerebellar granule cells (GCs, quirky) or Purkinje cells (PCs, purky) are sufficient to cause ASs in mice [20, 21].

The cerebellum has an extensive network of outputs originating from the CN, projecting to various thalamic nuclei as well as indirectly to cortical, and subcortical regions [22–28]. Therefore, in early animal studies electrical cerebellar cortical or CN stimulation was used to induce or stop various forms of epilepsy [29–33]. Based on these experimental approaches, several studies in human patients tried to harness the power of electrical cerebellar stimulation against epilepsy but with varying success [34–41]. All these studies used a fixed stimulation protocol independent of seizure occurrence. In contrast, more recent experimental approaches in rodents used closed-loop stimulation protocols to stop ongoing SWDs by transcranial or optogenetic stimulation of the neocortex or thalamus [42–44]. Based on these promising results, the cerebellar cortex and CN were stimulated during different types of ongoing seizures which proved to be successful [19, 45, 46]. Although optogenetic stimulation provided evidence for the role of the cerebellar circuit during various forms of seizures, little is known about the cellular and molecular underpinnings of this role, neither in terms of epileptogenesis nor in terms of treatment.

Here, we found that quirky and purky mice show recurrent bilateral SWDs with similar spectral characteristics and sensitivity to ETX as other mouse models of ASs, but with a more severe epileptic phenotype seen in purky mice. We used these models to study the activity of the CN and found that decreased activity in the CN was associated with the more severe phenotype of purky mice. Surprisingly, purky mice show an oscillatory pattern of action potential firing in the CN that is opposing to quirky and tottering mice [19]. We demonstrate that increasing CN activity via activation of the G_q_ signaling pathway using DREADDs [47] or the native metabotropic glutamate receptor 1 (mGluR1) specifically in the CN decreased the occurrence of SWDs. Furthermore, the application of a closed-loop light-stimulation system to activate CN neurons expressing the light-activated protein channelrhodopsin-2 (ChR2) [48] during ongoing SWDs stopped SWDs in both purky and quirky mice.

## Material and methods

### Animals

All data were collected in adult (12–52 weeks) mice of both sexes of the following lines: *Cacna1a*^*citrine*^/*Tg*^*Gabra−Cre*^ (Tg(Gabra6-cre)B1Lfr mice [stock #000196-UCD; B6;D2-Tg(Gabra6- cre)B1Lfr/Mmucd; [20, 49], *Cacna1a*^*citrine*^/*Tg*^*pcp2−Cre*^ (TgPcp2-cre mice [stock number 004146 B6.129-Tg(Pcp2-cre)2Mpin/J [50]*, Cacna1a*^*citrine*^ (as control for electrophysiological recordings in CN). While all mice were homozygous for *Cacna1a*^*citrine*^, the Cre allele was either homozygous or heterozygous in the used mice. The genetic background of each mouse was tested by PCR with genomic DNA from tail biopsy using the following primer pairs: *Cacna1a* forward 5′ GG GGTCTGACTTCTGATGGA 3′, reverse 5′ AAGTTGCACACAGGGCTTCT 3′; *Cacna1a*^*Citrine*^ forward 5′ TATATCATGGCCGACAAGCA 3′, reverse 5′ TTCGGTCTTCACAAGGAACC 3′; *Tg*^*Gabra6−cre*^ forward 5′ ATTCTCCCACCACCGTCAGTACG 3′, reverse 5′ AAAATTTGCCTG CATTACCG 3′; *Tg*^*pcp2−Cre*^ forward 5′ ATTCTCCCACCACCGTCAGTACG 3′, reverse 5′ AAAATTTGCCTGCATTACCG 3′. All animals were kept with a 12/12 day/night cycle and were solitarily housed with water and food ad libitum in individually ventilated cages.

### Virus production

The following protocol was used to produce recombinant adeno associated virus (AAV) virus stock of AAV-CMV-mCh, AAV-CaMkIIa-hM3D(G_q_).mCh (Addgene # 50476), AAV-CaMkIIa-hM4D(G_i__/o_).mCh (Addgene # 50477) and AAV-CaMKIIa-mCh. HEK 293T cells were cotransfected with the vector of interest, pAAV-RC9 and helper plasmid (pHelper) using polythylemine. Three days after transfection the cells were harvested, pelleted (300×*g* for 10 min 4 °C) and separated from the supernatant. The supernatant was mixed with 20% polyethylene glycol (PEG) and stored on a shaker at 4 °C for 2 h. The pellet was resuspended in 10 ml lysis buffer (0.15 M NaCl, 50 mM Tris–HCl, pH 8.5) and lysed by applying seven freeze/thaw cycles in dry ice/ethanol (15 min) and 37 °C water bath (10 min). Next, the lysate was incubated for 30 min at 37 °C with DNase I (20 mg/ml, Roche) and MgCl (1 M). Both the supernatant of the cells and the lysate were centrifuged at 3700×g for 20 min at 4 °C and the PEG-precipitated pellet was resuspended in the clarified cell lysate and stored overnight at 4 °C. The mixture was purified by adding 20% PEG for 2 h at 4 °C, centrifugated at 3700×g for 20 min at 4 °C and resuspended in 50 mM HEPES buffer. Next, room-temperature chloroform was added (ratio 1:1) and centrifuged at 370×g at RT for 5 min. The clear supernatant was collected using a syringe, filtered with a syringe filter (pore size 0.22 µm), 20% PEG was added and spun down at 3700×g for 20 min at 4 °C. Finally, the AAV was resuspended in 1 × PBS with 0.001% Pluronic F68, aliquoted and stored at − 80 °C. AAV2/1-hsyn-ChR2(H134R).eYFP (Addgene # 26973) and AAV1-hsyn-NpHR3.0.eYFP (Addgene # 26972) were obtained directly from the University of Pennsylvania (Vector core, Philadelphia, USA).

### Surgery

Mice were anesthetized with isoflurane (initial dose: 5% in 1.1 L/min air and 1.3–2.0% in 1.1 L/min air for maintenance), placed in a stereotaxic frame (Narishige, Tokyo, Japan) and received an s.c. injection of carprofen (2 mg/kg) for analgesia. During surgery, the pain reflex and the body temperature were controlled with a heating pad and to avoid corneal drying the eyes were coated with eye ointment. The skin was opened along the midline and the skull was exposed, cleaned and treated with Optibond All-in-One (Kerr GmbH, Rastatt, Germany). Five small craniotomies were used to insert ball tip goldwire electrodes (diameter: 0.15 mm; C.Hafner; Pforzheim, Germany) soldered to a microminiature connector (Digi-Key, Munich, Germany). Four electrodes were bilaterally implanted in the subdural space over the motor cortex (+ 1.5 mm AP, ± 1.5 mm ML relative to bregma) and somatosensory cortex (+ 1.5 mm AP, ± 3.0 mm ML relative to bregma). A grounding electrode was positioned on the lateral bone and an electrode that served as a reference was placed in the occipital bone (− 6.2 mm AP, 0 mm ML relative to bregma). For extracellular single-cell recordings or intracranial injections two additional craniotomies (diameter 3 mm) were made at AP (relative to bregma): − 6.2 mm ML: ± 2 mm using a microdrill. A recording chamber was constructed on the edge of the craniotomies with a light-curing hybrid composite (Charisma; Heraeus Kulzer, Hanau, Germany) and the tissue was covered with Kwik-Cast™ (World precision instruments, Sarasota, USA). All craniotomies were performed without damaging the dura mater. For head-fixation during extracellular single-cell recordings or intracranial injections, a pedestal was secured lateral to the middle of the head (after embedding the electrodes with dental acrylic). In all mice, the incision was closed by embedding the electrodes with a light-curing hybrid composite (Charisma; Heraeus Kulzer, Hanau, Germany) and dental acrylic. Mice were allowed to recover for at least 7 days before habituation or recordings. Mice that underwent head-fixation, were habituated to the setup for 3–5 consecutive days (30–60 min/day) prior to the first recording.

### Virus injection and optical fiber implantation

For optogenetic or chemogenetic experiments, viral injections in the CN were performed before implanting the ECoG electrodes. Two small craniotomies (relative to bregma: − 6.2 mm AP, ± 2 mm ML) were made and a custom-made glass pipette attached to a 5-ml syringe was lowered (from the surface of the brain) to 1.8–2.3 mm DV in quirky and due to the severe cerebellar degeneration to − 1.5 to 1.9 mm DV in purky mice. On each side, 200– 250 nl virus was injected in 100 µm intervals using pressure injection. For optogenetic stimulation bilateral custom-made optical fibers (ferrule 1.25 mm outer diameter, 230 µm bore size (CFLC230-10); fiber diameter 200 µm, 0.39 NA, FT200EMT; Thorlabs, Dortmund, Germany) were bilaterally inserted into the craniotomy and perpendicularly secured at − 1.8 mm and -1.5 mm (for quirky and purky mice, respectively) with a light-curing hybrid composite (Charisma; Heraeus Kulzer, Hanau, Germany). After injection, the ECoG electrodes were implanted as described. Mice were allowed to recover for at least 2 weeks before recordings were started to ensure sufficient viral expression.

### Electrophysiological recordings

The ECoG and extracellular single-cell activity was recorded using Spike 2 software (version 7; Cambridge Electronic Design Ltd; Cambridge, UK). ECoG signals were amplified by 5000 and bandpass filtered (3–100 Hz for ECoG signals; (DPA-2FX, npi electronic GmbH; Tamm, Germany) and sampled at 400 Hz (CED 1401, Cambridge Electronic Design Ltd; Cambridge, UK). For extracellular single-cell recordings, mice were awake and head-fixed but able to move all limbs freely on a treadmill. Before recording the Kwik-Cast™ was carefully removed and the craniotomies were cleaned. Custom made borosilicate glass capillaries (OD 1.5 mm, ID 0.86, resistance 3–5 MΩ BF150-86-10, Science products, Hofheim, Germany)) filled with 2 M NaCl were used to advance through the open craniotomies using a micromanipulator (MPC 200, Sutter Instruments, Novato, USA). CN neurons were identified based on stereotactic coordinates, recording depth (beginning at 1800 µm in quirky and 1500 µm in purky) and characteristic density of neurons. Signals were bandpass filtered 0.3–3 kHz, amplified 2000-4000 (EXT-02F/1, npi electronic GmbH; Tamm, Germany) and sampled at 20 kHz (CED 1401, Cambridge Electronic Design Ltd; Cambridge, UK). Recordings lasted no longer than 4 h per day. ECoG activity was recorded bilaterally in S1 during extracellular recordings.

### Pharmacological modulation of spike-wave discharges

Before drug injection mice were recorded for 1.5 h (30-min habituation and 1 h baseline; pre). Drugs were dissolved in 0.2 ml of their corresponding vehicle solution: saline (ethosuximide (Sigma-Aldrich)), saline + 1% Triton-X (valproic acid), saline + 5% dimethyl sulfoxide (clozapine N-oxide (Tocris)). Control injections consisted of 0.2 ml of the corresponding vehicle solution after 1.5 h. After injection ECoG signals were recorded for 1 h (post). Before intracranial (ic) injection Kwik-Cast™ was carefully removed from the craniotomy to expose the brain. A borosilicate glass capillary was filled with ~ 0.4 μl of the drug (50 mM (S)-3,5-dihydroxyphenylglycine (Tocris) in saline or 14 mM TCB-2 (Tocris) in saline) connected to a 5 ml syringe. With the help of a micromanipulator (MPC 200, Sutter Instruments, Novato, USA) the micropipette was carefully advanced through the dura until a depth of 1800 µm (quirky) or 1500 μm (purky) was reached. The micropipette was withdrawn slowly after pressure injection and the ECoG signals were recorded for another 1 h.

### Optogenetic stimulation of CN neurons

Optogenetic stimulation of the CN neurons was performed using a 473 nm solid-state laser (Crystalaser 473 nm, Reno, USA) or two 465 nm compact LED modules (PlexBright Compact LED Module 465 nm, Plexon, Texas, USA). For halorhodopsin and orange light control stimulation two 620 nm compact LED modules (PlexBright Compact LED Module 620 nm, Plexon, Texas, USA) were used. During closed-loop stimulation a threshold was manually set based on the individual SWD amplitude in each animal in the ECoG. Each closed loop protocol consisted of two different sequences that were given randomly with a chance of 50%. Once the threshold was passed either a 1 s (100 ms, 50 ms, 30 ms or 10 ms) light stimulus was given bi- or unilaterally with a delay of 100 ms or no light was given at all (sham; internal control to verify threshold level and efficiency of stimulation per animal). To avoid continuous stimulation, a delay of 10 s followed each light pulse. At the end of the experiment, the laser or LED output at the optogenetic patch cable was measured with a photodiode (~ 0.5–1 mW at the tip of the fiber; corresponding to ~ 3.98–7.95 mW/mm^2^ directly underneath the tip). For unilateral stimulation, the optic fiber on one side was connected to the optic cable.

### Seizure definition and analysis

Offline analysis of recorded digital signals was performed manually using a custom written Matlab script. For incidence of SWDs per hour, events were considered as SWDs if the following criteria for each SWD were fulfilled: 1) a bilateral occurrence 2) a voltage amplitude exceeding the baseline fluctuation twice, and 3) duration of ≥ 0.5 s/event. The duration of SWDs was defined as the time between the first appearance of a spike in the primary motor cortex or the primary somatosensory cortex and the natural termination defined as the last wave without further spikes within 0.5 s. Spectral analysis of ECoG signals was performed with a fast Fourier transform (FFT) algorithm with the help of a custom written Matlab script. A single Blackman window and 512 (frequency) or 1024 (peak frequency) nodes corresponding to a temporal resolution of 1.28 s or 2.56 s (peak frequency) and a spectral resolution of 0.78 Hz or 1.54 Hz (peak frequency) were analyzed. For temporal precise spectral analysis, a complex morlet wavelet analysis with a bandwidth of 0.1 s and a central frequency of 6 Hz was used. Spectrograms were normalized to the maximum power value. Optogenetic induced termination of SWDs was calculated by comparing the seizure characteristic spectral power (quirky Hz 5.4–9.3 Hz, purky: 3.1–7.8 Hz) by FFT before stimulation (pre) and after stimulation (post). All signals were included if they consisted of at least two consecutive SWD peaks before stimulation and did not terminate naturally before onset of stimulation. Efficiency of stimulation and latency until SWDs stopped were calculated by manually dividing SWDs into stopped and continued based on the presence of SWD peaks after stimulation (≤ 2, stopped; > 2, continued) before the ECoG returned to baseline activity. To analyze the effect of NpHR stimulation on SWD duration mean duration of SWDs per animal pre and post stimulation was normalized to the mean duration of SWDs pre and post Sham stimulation.

### Analysis of electrophysiological data

Single-cell recordings were included if the activity was well isolated and stable (at least 100 s). Individual action potentials were detected using a threshold-based custom-written Matlab script and the firing rate, coefficient of variation of inter-spike intervals (ISIs) = σ_ISI_/μ_ISI_, CV2 = 2|ISIn + 1− ISIn|/(ISIn + 1 + ISIn) and burst index = number of action potentials within bursts (≥ 3 consecutive action potentials with an ISI ≤ 10 ms)/ total number of action potentials were calculated.

For analysis of action potential firing during SWDs individual SWD peaks of the SWDs were manually selected in S1 ECoG recordings using a threshold. A mean ECoG peak was calculated individually for each selected SWD using a duration of 200 ms surrounding all SWD peaks (100 before and 100 ms after the peak). For each mean ECoG peak the corresponding perievent time histogram (PETH) of action potentials was calculated with a bin size of 5 ms and a total duration of 200 ms (100 ms before and 100 ms after the peak). The PETH was used to calculate an individual modulation amplitude for each cell by subtracting the number of action potentials during/before the spike (− 30 to 5 ms in the PETH) and during the wave (25 to 60 ms in the PETH) the maximum amplitude of the SWD. To generate a normal distribution for the modulation amplitude the ISIs of PETH were randomly shuffled 1000 times. For each shuffled data set, a new PETH was calculated using the same bin size and duration and a new modulation amplitude was calculated. Next, a Z-score was generated using the shuffled modulation amplitudes and the original modulation amplitudes by Z-score = (modulation amplitude – μ_modulation amplitude-shuffled_) / σ_modulation amplitude-shuffled_ μ_modulation amplitude-shuffled_ is defined as the mean modulation amplitude for the shuffled data and σ_modulation amplitude shuffled_ defined as the standard deviation for the shuffled data. A cell was considered seizure modulated if the Z-score of the modulation amplitude was lower than − 1.96 (quirky) or higher than − 1.96 (purky) corresponding to a *p* value > 0.05.

### Histology, antibody staining and cell counting

After the last recording session, mice were sacrificed for histological verification of correct viral expression. Mice were anesthetized and transcardially perfused with phosphate-buffered saline (pH 7.4, PBS) and 4% Paraformaldehyde (PFA; Sigma Aldrich, Taufkirchen, Germany) in PBS. Dissected brains were post fixed for 1–3 h in 4% PFA and cryoprotected in 30% sucrose (wt/v; Sigma Aldrich, Taufkirchen, Germany) in PBS at 4 °C for 2 days. Forty-µm-thick parasagittal or coronal sections were collected in PBS and permeabilized for 30 min in 0.3% PBS-T (0.3% Triton-X (v/v) in PBS) for Nissl staining and permeabilized and blocked with 1–3% serum in 0.3% PBS-T for immunofluorescent labeling. Selected sections were then stained against Nissl by incubation in PBS with either 640/660 or 435/455 Neurotrace (1:300; Thermo Fisher Scientific, Waltham, USA) or with the following primary and secondary antibody pairs mouse anti parvalbumin (1:1000, Sigma Aldrich, Taufkirchen, Germany), Dylight 650 donkey anti mouse (1:500, Abcam, Berlin, Germany) and mouse anti Cre (1:500, Millipore, Darmstadt, Germany), Alexa Flour 568 donkey anti mouse (1:1000, Life technology cooperation). All antibodies were diluted 1:300–1000 and incubated overnight at 4 °C and then washed and incubated with secondary antibodies for 2 h at room temperature. After washing in PBS brain sections were embedded in Roti®mount Fluocare (CarlRoth, Karlsruhe, Germany). Images were captured using a Leica TCS SP5II (LAS AF 2.6) inverted confocal laser scanning microscope (Leica DMI6000 B, Wetzlar, Germany) with a 20x/0.7 NA and 40x/0.6 NA objective. For excitation 405 nm (Neurotrace 435), 488 nm (eGFP), 514 nm (eYFP), 561 nm (mCh) and 630 nm (Neurotrace 640) were used. Images were later analyzed using ImageJ software. For cell counting pictures with magnification of 40 × were taken for three areas (Crus I, Lobe 3 and Lobe 10) in each animal and the number of Cre negative (only Nissl expression) and Cre positive cells (Nissl and Cre expression) were counted.

### Statistics

Statistical significance, test procedure and number of cells/animals and/or trials performed (*n*) are specified in the figures and/or figure legends. Statistical significance in all experiments was evaluated using SigmaPlot software v12.5 (Systat Software). Normality of distributions was tested using Shapiro–Wilk tests. For all results, the level of significance was set to *P* < 0.05. Statistical significance is indicated with *** *p* < 0.001; ** *p* < 0.01; * *p* < 0.05; n.s. (not significant) and reported in figure legends.

## Results

### Quirky and purky mice show differences in seizure occurrence, duration and sensitivity to anti-epileptic drugs

We previously described that conditional knockout of the P/Q-type calcium channel in cerebellar GCs as well as PCs can be responsible for a pathological phenotype that resembles classical rodent models of P/Q-type calcium channel malfunction including ataxia, dystonia and ASs [20, 21]. However, little is known about the characteristic features of SWDs in these new rodent models. Therefore, we characterized the occurrence, spectral features, duration and responsiveness to commonly prescribed anti-epileptic drugs for SWDs in quirky and purky mice by recording bilateral electrocorticogram (ECoG) activity in the primary motor (M1) and somatosensory cortex (S1).

**Fig. 1:**
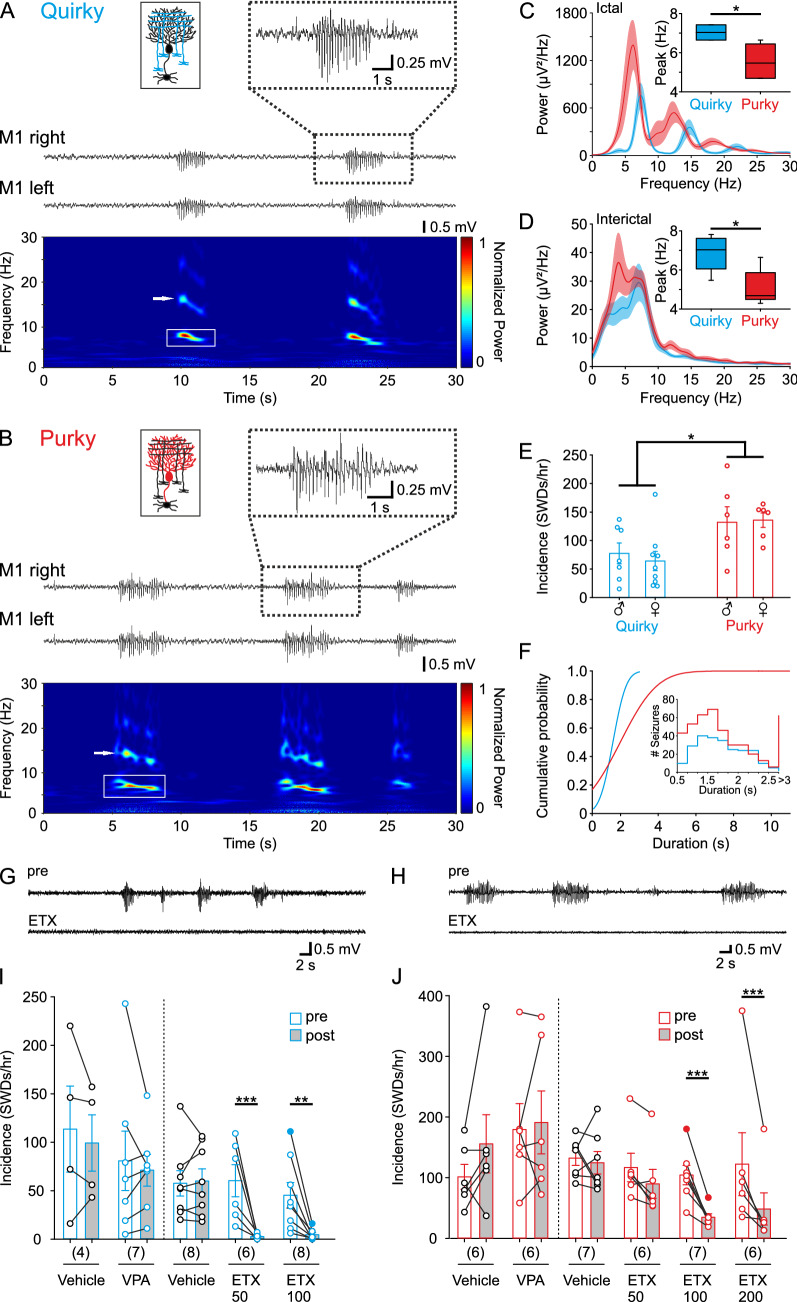
Quirky and purky mice show differences in seizure occurrence, duration and sensitivity to anti-epileptic drugs. **A** and **B** Mouse lines with a restricted knockout of the P/Q-type calcium channel in (**A**) cerebellar granule cells (quirky; cyan) and (**B**) Purkinje cells (purky; red) display typical absence seizures that can be detected bilaterally by ECoG recordings. Wavelet analysis showed the main frequency between 5–7 Hz (white box) with corresponding harmonics (white arrow). **C** and **D** Ictal and interictal spectral power for quirky (*n* = 5; mean of 40 SWDs per animal was used) and purky mice (*n* = 5; mean of 40 SWDs per animal was used). Inlet depicts the peak frequency, which is significantly higher in quirky than purky mice for ictal (two-sided Mann–Whitney *U* test) and interictal (two-sided Mann–Whitney *U* test) activity. Thick lines represent mean power and shadowed areas represent ± SEM. Boxplot depicts median, 25th and 75th percentile and whiskers 5th and 95th percentile. **E** No difference was found between male (quirky *n* = 7; purky *n* = 6) and female (quirky *n* = 9; purky *n* = 6) in the incidence of SWDs but quirky mice showed significantly fewer SWDs per hour than purky mice (two-way ANOVA with pairwise Bonferroni test). Bars represent mean ± SEM and individual animals are represented as circles. **F** Quirky mice showed shorter SWDs than purky mice as depicted by the cumulative probability for SWD duration during 1 h recording (*n* = 4 per mouse line). Inlet depicts the number of SWDs for increasing duration increments. **G** and **H** Example recording of (**G**) quirky and (**H**) purky mice before and after injection of ethosuximide (ETX). Examples are expressed as filled circles in **I** and **J**. **I** and **J** Quirky and purky mice show no sensitivity to valproic acid but are sensitive to ETX (two-way repeated measure ANOVA with pairwise Bonferroni test). White bars represent the mean incidence of SWDs 1 hour before injection (pre) and 1 hour after injection (grey bar; post). Vehicle injection is depicted with black circles. Bars represent mean ± SEM and individual animals are represented as circles. For detailed statistical analysis, see Supplementary Table 1. *p value < 0.05; **p value < 0.01; ***p value < 0.001

Quirky and purky mice displayed spontaneous bilateral 5–7 Hz SWDs with concomitant behavioral arrest and without diurnal cycle (Fig. 1A–D, Supplementary Fig. 1). Spectral analysis showed a slightly higher initial frequency which slowly decreased towards the end (Fig. 1A, B). Analysis of ictal as well as interictal activity depicted a peak at a significantly higher frequency for quirky mice in comparison to purky mice (Fig. 1C, D; ictal peak quirky 7.0 ± 0.1 Hz, purky 5.5 ± 0.4 Hz). We found no significant difference in the occurrence of SWDs between male (quirky 77.4 ± 16.9 SWDs/hr, purky 132.2 ± 24.7 SWDs/hr) and female (quirky 64.1 ± 15.9 SWDs/hr, purky 135.8 ± 11.5 SWDs/hr) mice for both lines, therefore we used both male and female mice for the following experiments (Fig. 1E). However, purky mice showed significantly more SWDs per hour which lasted longer on average than in quirky mice (quirky 1.60 ± 0.04 s, purky 1.99 ± 0.07 s, Fig. 1E and F). One of the characteristic features of SWDs is the sensitivity to ETX as well as valproic acid (VPA) [3]. Intraperitoneal (ip) injection of 200 mg/kg VPA did not change occurrence of SWDs in comparison to baseline recordings. In contrast, ETX injection significantly reduced the occurrence of SWDs for 50 mg/kg and 100 mg/kg in quirky as well as 200 mg/kg in purky mice (Fig. 1G–J).

Surprisingly, knockout of the P/Q-type calcium channel in cerebellar GCs in another mouse line does not result in the described phenotype (α^6^cre-Cacna1a KO) [51]. Therefore, we tested whether the expression of Cre recombinase in cerebellar granule cells differs from α^6^cre-Cacna1a KO. In contrast to α^6^cre-Cacna1a KO where 14–25% of GCs lack Cre expression we found 5% of GCs lack Cre expression in quirky mice (Supplementary Fig. 2). Overall, quirky and purky mice show characteristic features that can be found in rodent models of ASs but with distinct differences between these two lines. This makes quirky and purky mice suitable murine models to understand the impact of the cerebellum on the generation, maintenance and pharmacological sensitivity of SWDs in mice.

### CN neurons fire aberrantly and seizure-modulated in quirky and purky mice

**Fig. 2:**
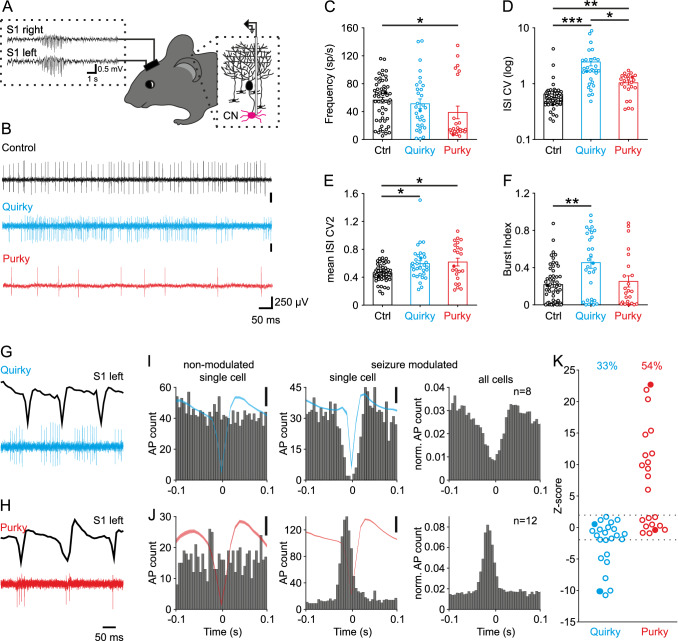
CN neurons fire aberrantly and seizure-modulated in quirky and purky mice. **A** Experimental design: Extracellular recordings of CN (magenta) neurons were performed in awake head-restrained mice with simultaneous ECoG recordings. **B** Example of CN neuron activity in control (black), quirky (cyan) and purky (red) mice. Examples are expressed as filled circles in (**C** to **F)**. **C** Mean firing rate, **(D)** interspike interval coefficient of variation and **(E)** mean interspike interval coefficient of variation 2 and **(F)** burst index of CN neurons in control (*n* = 53 recorded from eight mice), quirky (*n* = 31 recorded from eight mice) and purky (*n* = 23 recorded from three mice) mice. **C** to **F** bars represent mean ± SEM and individual cells are represented as circles (Kruskal–Wallis test with pairwise Dunn’s test). **G** and** H** Example of (**G**) quirky and (**H**) purky CN neurons that are phase-locked to the ictal activity. **I** and **J** Raster plot of non-modulated and seizure-modulated CN neuron with corresponding mean spike-and-wave complex. Scale bar represents 0.1 mV. Single cell examples are expressed as filled circles in **K**. **I** Quirky mice show a decrease in action potential firing during the spike of the spike-and-wave complex while (**J**) purky mice show an increase in action potential firing. Thick lines represent mean spike-and-wave complex activity and shadowed areas represent ± SEM. **K** Proportion of CN neurons in quirky (*n* = 23 recorded from seven mice) and purky (*n* = 22 recorded from three mice) mice that fire phase-locked with the ictal activity (quirky 33% and purky 54%). The horizontal dotted line represents ± 1.96 (corresponding to *p* < 0.05). For detailed statistical analysis see Supplementary Table 2. *p value < 0.05; **p value < 0.01; ***p value < 0.001

Since the conditional knockout of the P/Q-type calcium channel in two different cerebellar cell types resulted in similar behavioral changes we tested whether the cerebellar output is affected. CN neurons are not directly affected by the knockout of the P/Q-type calcium channel, but we hypothesized that their firing properties are indirectly altered by the changes upstream in the cerebellar cortex. We extracellularly recorded CN neuronal firing patterns (lateral nucleus) and simultaneously ECoG activity in M1 from awake head-restrained mice, since activity in the CN is known to be modulated during SWDs (Fig. 2A, B) [19, 52]. CN neurons of quirky and control mice displayed similar mean firing frequencies (control 56.4 ± 4.1 Hz, quirky 51.2 ± 6.8 Hz) while purky CN neurons fired significantly slower (purky 38.8 ± 8.9 Hz, Fig. 2C). Both mutant mouse lines showed a more irregular firing pattern with interrupted and bursting bouts indicated by an increased CV, CV2 and burst index (Fig. 2D, F). Further analysis of CN activity during SWDs revealed two different types of neurons, non-modulated and seizure-modulated neurons in quirky and purky mice (Fig. 2G, H). Seizure non-modulated neurons did not show a pattern corresponding to the phase of the mean cortical oscillation (Fig. 2I, J). In contrast, seizure-modulated neurons in quirky mice showed a decrease in firing during the spike phase of the SWD, whereas in purky mice the seizure-modulated neurons increased firing during the spike phase of the SWD. Overall, in quirky 33% and purky 54% of CN neurons showed a seizure-modulated firing pattern (Fig. 2K). Taken together, CN neurons in quirky and purky mice show decreased firing precision and increased action potential firing in their seizure-modulated neurons during the wave in quirky and during the spike in purky mice.

### Transient activation or silencing of CN neurons decreases or increases SWD occurrence in quirky and purky mice

**Fig. 3:**
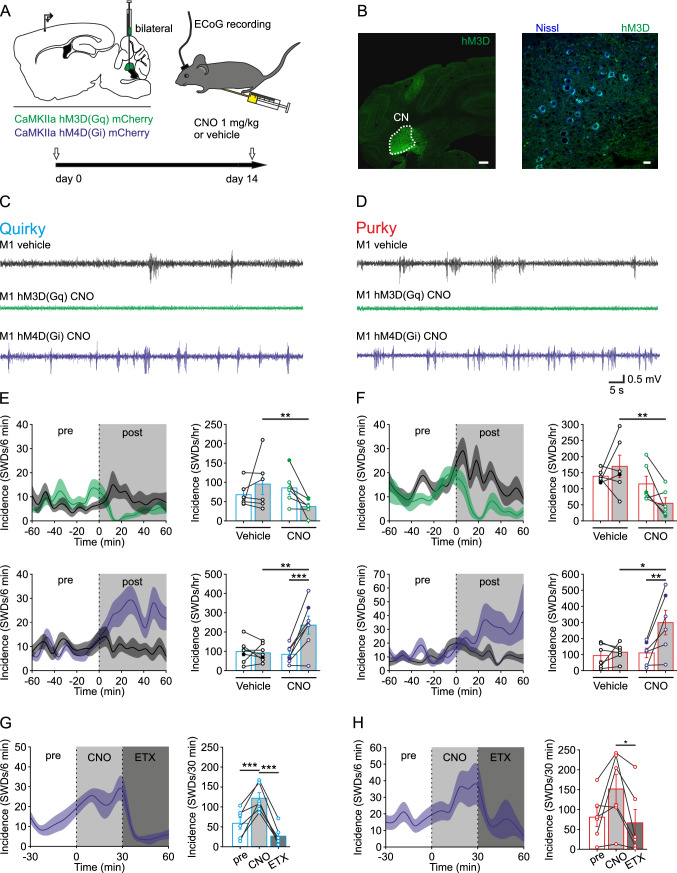
Transient activation or silencing of the CN neurons decreases or increases SWD occurrence in quirky and purky mice. **A** Experimental design: Experiments were started 14 days after bilateral AAV injection. After 1 hour of baseline recording, mice were injected ip with 1 mg/kg clozapine N-oxide (CNO) or vehicle. **B** Representative example of hM3D (DREADD G_q_) expression in the CN in a coronal section of a quirky mouse. Scale bar 100 µm and 10 µm. **C** and **D** Representative example of ECoG activity (S1 left) after vehicle (black) or CNO injection in (**C**) quirky (cyan) and (**D**) purky (red) mice expressing hM3D (DREADD G_q_, green) or hM4D (DREADD G_i/o_, dark blue) in the CN. Examples are expressed as filled circles in **E** and **F**. **E** and **F** Change in the incidence of SWDs before (pre, white box) or after (post, grey box) injection of vehicle or CNO in (**E**) quirky (vehicle *n* = 6/7, hM3D *n* = 6, hM4D *n* = 7) and (**F**) purky (vehicle *n* = 6/6, hM3D *n* = 6, hM4D *n* = 6) mice. Thick lines represent mean incidence and shadowed areas represent ± SEM. Number of SWDs before (white bar) and after injection of vehicle or CNO in quirky and purky mice. After injection of CNO quirky and purky mice expressing hM3D showed significantly fewer SWDs and mice expressing hM4D significantly more (two-way repeated measure ANOVA with pairwise Bonferroni test). **G** and **H** Change in the incidence of SWDs after ip injection of 1 mg/kg CNO (light grey box) and subsequent ip injection of 100 mg/kg ethosuximide (dark grey box) in (**G**) quirky (*n* = 6, cyan) and (**H**) purky (*n* = 6, red) mice expressing hM4D (G_i/o_ DREADD) in the CN. Thick lines represent mean incidence and shadowed areas represent ± SEM. Number of SWDs before (white bar), after CNO injection (light grey bar) and after subsequent ETX (dark grey bar) injection in quirky (one-way repeated measures ANOVA with all pairwise Bonferroni test) and purky (one-way repeated measures ANOVA with all pairwise Bonferroni test) mice. Bars represent mean ± SEM and individual animals are represented as circles. For detailed statistical analysis, see Supplementary Table 3. *p value < 0.05, **p value < 0.01, ***p value < 0.001

Since purky mice showed a more severe phenotype with longer and more frequently occurring SWDs, we hypothesized that the decreased CN firing and the accompanying lower excitatory drive might be responsible for the more severe epileptic phenotype seen in purky mice. Thus, we tested if transient activation of the CN can reduce the number of SWDs in quirky and purky mice and vice versa if a decrease in CN activity could increase the number of SWDs. We expressed the excitatory DREADD hM3D (G_q_) and the inhibitory DREADD hM4D (G_i/o_) in CN neurons using the Ca^2+^/calmodulin dependent protein kinase II (CamK2) promoter and recorded ECoG activity 14 days after AAV injection (Fig. 3A, B). As a control, we expressed the fluorescent protein mCherry (mCh) and verified that injection of 1 mg/kg clozapine N-oxide (CNO) alone did not affect the number of SWDs in quirky and purky mice (Supplementary Fig. 3). Next, we recorded 1-hour baseline activity and injected 1 mg/kg CNO or vehicle ip followed by a 1-hour ECoG recording (Fig. 3C–F). We found that 1 mg/kg CNO in mice expressing the excitatory DREADD significantly reduced the occurrence of SWDs while activation of the inhibitory DREADD significantly increased the occurrence of SWDs in quirky and purky mice (Fig. 3C, D). After CNO injection mice expressing the excitatory DREADD hM3D (G_q_) had a decreased motor activity as a result of the broad activation of the G_q_ receptor. Since typical SWDs can be controlled using ETX (Fig. 1G, H), we tested whether the increased occurrence of SWDs after CNO injection in animals expressing the inhibitory DREADD can also be controlled by ETX. Interestingly, ETX was able to reduce the occurrence of SWDs even after the previous increase due to the inhibitory DREADD (Fig. 3G, H). Together, artificial increase in CN activity has an anti-epileptic potential, whereas artificial decreased CN activity is pro-epileptic in quirky and purky mice.

### Modulation of mGluR1 signaling in CN neurons can control SWDs in quirky and purky mice

**Fig. 4:**
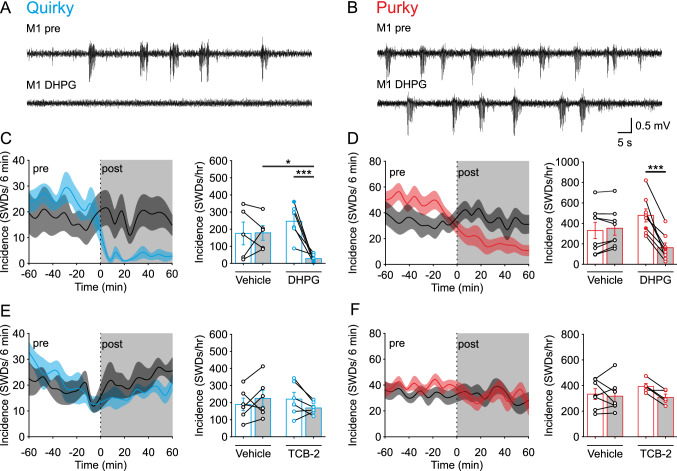
Modulation of mGluR1 signaling in the CN neurons can control SWDs in quirky and purky mice. **A** and **B** Representative example of ECoG activity (M1 left) before or after injection of the metabotropic glutamate receptor (mGluR) 1/5 agonist dihydroxyphenylglycin (DHPG) in the CN in (**A**) quirky and (**B**) purky mice. Examples are expressed as filled circles in **C**–**D**. **C** and **D** Change in the incidence of SWDs after (grey box) direct CN injection of vehicle (black, *n* = 5) or DHPG (*n* = 6) in (**C**) quirky (cyan) and (**D**) purky (red, vehicle *n* = 6, DHPG *n* = 5) mice. Number of SWDs after DHPG injection decreased in comparison to vehicle injection in quirky and purky mice (two-way repeated measure ANOVA with pairwise Bonferroni test). **E** and **F** Change in the incidence of SWDs after direct CN injection of vehicle (black, *n* = 6) or TCB-2 (*n* = 6) in (**E**) quirky and (**F**) purky (red, vehicle *n* = 7, TCB-2 *n* = 5) mice. Thick lines represent mean incidence and shadowed areas represent ± SEM. Bars represent mean ± SEM and individual animals are represented as circles. For detailed statistical analysis see Supplementary Table 4. *p value < 0.05, ***p value < 0.001

Our G_q_-specific DREADD experiments (Fig. 3) implicate that the G_q_-coupled pathway is a potential anti-epileptic mechanism for controlling ASs from the cerebellum. Therefore, we decided to investigate the role of the mGluR1, a G_q_-coupled receptor that is highly expressed in the CN [53] using the mGluR1/5 specific agonist dihydroxyphenylglycine (DHPG). Furthermore, mGluR1 activation is known to have antiepileptic properties when activated systemically, intrathalamically or intracortically in a rat model of ASs [54, 55]. Unilateral injection of DHPG in the CN significantly decreased the occurrence of SWDs in head-fixed quirky and purky mice compared to baseline (Fig. 4A–D). Following injection of DHPG, mice often displayed an increased facial grooming/washing as described for DHPG injections [56].

To test whether the antiepileptic effect was limited to mGluR1 activation, we injected the agonist for the 5-HT2A receptor, another G_q_-coupled GPCR that is highly expressed in the CN [57, 58]. Although the antiepileptic effect of TCB-2 was already demonstrated after systemic injection [59], we found no change in the incidence of SWDs following CN injection in quirky and purky mice (Fig. 4E, F). Note, we found that quirky and purky mice show an elevated incidence of SWDs during head-fixation in comparison to freely moving animals which we used during all other experiments (Supplementary Fig. 4). These data demonstrate that activation of native mGluR1 receptors inside the CN have an anti-epileptic effect in both quirky and purky mice.

### SWDs can be stopped by single-pulse stimulation of the CN in quirky and purky mice

**Fig. 5:**
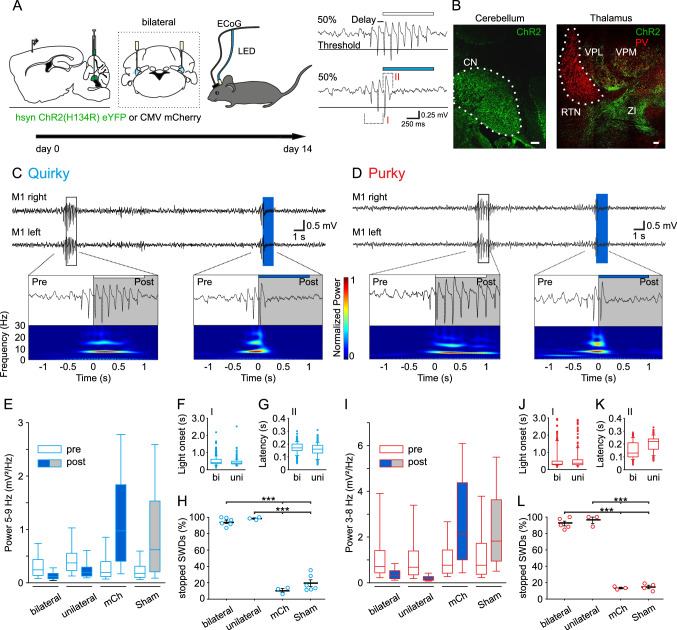
SWDs can be stopped by single-pulse stimulation of the CN in quirky and purky mice. **A** Experimental design: Experiments were started 14 days after bilateral AAV injection in the CN in quirky and purky mice. During recordings, an individual threshold was used to quickly identify SWDs. Sham (no light) or blue (465 nm, blue box) light stimulation was triggered with a 50/50 chance after a 100 ms delay for 1 s if the threshold was crossed. **B** Representative example of Channelrhodopsin2 (ChR2, green) expression in a parasagittal slice of the CN and expression of axonal terminals inside thalamic nuclei in a quirky mouse. Reticular thalamic nucleus (RTN), ventral posterolateral thalamic nucleus (VPL), ventral posteromedial thalamic nucleus (VPM), zona incerta (ZI), parvalbumin (PV). Scale bar 100 µm. **C** and **D** Representative example ECoG activity and power spectrum analysis before (pre, white rectangle) and after (post, grey rectangle) 1 s sham (no stimulation, small white box) or blue light ChR2 stimulation (small blue box) of CN neurons during ongoing SWDs in (C) quirky (cyan) and (D) purky (red) mice. **E** and **I** Boxplots represent median SWD-specific power before (white boxplot) and after (blue and grey boxplot) bilateral (*n* = 1104 from six animals) or unilateral (*n* = 312 from three animals) ChR2 stimulation and mCherry (mCh, *n* = 317 from three animals) or Sham (*n* = 819 from six animals) stimulation in (E) quirky and (I) purky mice (bilateral (*n* = 377 from five animals), unilateral (*n* = 379 from three animals), mCh (*n* = 299 from three animals) or Sham (*n* = 261 from four animals)) mice. SWD-specific power was decreased after bilateral and unilateral stimulation and not after mCh or Sham stimulation in quirky and purky mice. **F** and **J** Delay until light onset after first electrographic spikes (see A, inlet I) was 506 ms before bilateral (*n* = 326 from four animals) or 443 ms before unilateral (*n* = 291 from three animals) stimulation in (**F**) quirky and 590 ms before bilateral or 581 ms before unilateral stimulation in (**J**) purky mice. **G** and **K** Latency until SWDs stopped after light onset (see A, inlet II) was 169 ms for bilateral (*n* = 326 from four animals) or 157 ms for unilateral (*n* = 291 from three animals) stimulation in (G) quirky and 153 ms for bilateral (*n* = 247 from four animals) or 204 ms for unilateral (*n* = 260 from three animals) stimulation in (**K**) purky mice. Boxplot depicts median, 25th and 75th percentile and whiskers 5th and 95th percentile. Individual SWDs are represented as circles. **H** and **L** Percentage of successfully stopped seizures for bilateral (*n* = 6) and unilateral (*n* = 3) light stimulation was significantly higher than mCh (*n* = 3) or Sham (*n* = 6) stimulation in (H) quirky and (L) purky mice (bilateral *n* = 5, unilateral *n* = 3, mCh *n* = 3, Sham *n* = 4, one-way ANOVA). Lines represent mean ± SEM and individual animals are represented as circles. For detailed statistical analysis, see Supplementary Table 5. ***p value < 0.001

Based on the changes in CN neuronal firing during SWDs, we hypothesized that excitatory output from the CN can break the oscillatory cycle and thus stop the ictal activity. Therefore, we expressed ChR2, a light-sensitive cation-channel, under the control of the pan-neuronal human synapsin 1 gene (hsyn) promoter in the CN and recorded ECoG activity 14 days after AAV injection (Fig. 5A). During ECoG recordings, light stimulation was automatically triggered after a delay of 100 ms if the SWD spike exceeded an individually set threshold. As an internal control, light was triggered only in 50% of all trials, while no light was given for the remaining trials (sham stimulation). To test if visible light and emitted heat alone affected SWDs, mCh alone was expressed in CN neurons of quirky and purky mice and bilaterally stimulated with blue light. Bilateral light stimulation of ChR2 in CN neurons during SWDs stopped ongoing SWDs in quirky and purky mice as indicated by the power decrease in the SWD-specific frequency (Fig. 5C, D right), while sham stimulation did not change the SWD (Fig. 5C, D, left). Repetitive SWD disruption was robust in all tested animals and even unilateral CN stimulation was equally successful in stopping SWDs but not in mCh expressing or sham stimulated mice (Fig. 5E, I).

We also examined whether optogenetic silencing of CN using NpHR a light-sensitive chloride pump could enhance the duration of SWDs. However, NpHR stimulation with orange light did not change the mean duration of SWDs (Supplementary Fig. 5). To understand the temporal dynamics of SWD stopping by ChR2 stimulation, we analyzed the duration of the first peak of the SWD until the onset of light stimulation and latency of SWD recovery. We found stimulation started between 440 and 505 ms after the first peak of the SWD in quirky (quirky bilateral 506.2 ± 15.0 ms and quirky unilateral 442.9 ± 11.7 ms, Fig. 5F) and between 550 and 590 ms after the first peak of the SWD in purky (purky bilateral 590.2 ± 39.7 ms and purky unilateral 581.3 ± 36.0 ms, Fig. 5J) mice. SWDs that were stimulated with blue light in ChR2 expressing animals returned to baseline after ~ 155–170 ms in quirky (quirky bilateral 169.5 ± 2.3 ms quirky unilateral 156.6 ± 2.8 ms, Fig. 5G) and after ~ 150–200 ms in purky (purky bilateral 153.3 ± 4.0 ms, purky unilateral 203.6 ± 3.8 ms, Fig. 5K) mice. Since the activity returned to baseline after less than 1 s we decided to test if shorter light pulses (100 ms, 50 ms, 30 ms and 10 ms) were equally effective. Indeed, 100 ms, 50 ms and 30 ms successfully stopped SWDs, whereas 10 ms only partially stopped SWDs (Supplementary Fig. 6). As a control the same mice were stimulated with 620 nm (orange) light pulses for 100 ms which does not activate ChR2, and no change in SWDs was evident. The effectivity of CN stimulation to cease further SWD formation was 95–100% in quirky (quirky bilateral 95%, quirky unilateral 100%, quirky mCh 11%, quirky sham 19%, Fig. 5H) and 92–96% in purky (purky bilateral 92%, purky unilateral 96%, purky mCh 13% and purky sham 15%, Fig. 5L) mice. SWDs that stopped during mCh or sham stimulation were considered naturally stopped. Taken together, brief optogenetic stimulation of the CN can effectively terminate ongoing SWDs in quirky and purky mice in approximately 200 ms.

## Discussion

We characterized two different AS mouse models both harboring a conditional knockout of the P/Q-type calcium channel in a different subset of cerebellar cells and found that chemogenetic, pharmacologic and optogenetic stimulation of the CN can control the occurrence as well as continuation of SWDs in the neocortex. Furthermore, we found that specifically low activity in the CN correlates with a more severe epileptic phenotype. By chemogenetically or pharmacologically increasing CN activity via the G_q_ pathway we could decrease SWDs. Our two mouse models provide evidence that changes inside the cerebellar circuit can lead to the manifestation of SWDs and thus are viable models to study the underlying circuit alterations that arise in the thalamo-cortical network (TCN). Furthermore, our results provide evidence that targeted therapy that is not restricted to the classical seizure network can have beneficial effects which opens further research for therapies that do not target the classical seizure network.

### Quirky and purky mice as models for absence seizures

The number of SWDs is variable among different rodent models of ASs and in general rat models show fewer but longer SWDs than mouse models [12, 60, 61]. Our data from quirky and purky mice are comparable to the data reported for other mouse lines regarding SWD frequency, occurrence as well as duration, and show that quirky and purky mice are suitable models to understand the cerebellar contribution to ASs [60, 62]. Interestingly, two other mouse models harboring a conditional knockout of the P/Q-type calcium channel in cerebellar GCs (α^6^cre-Cacna1a KO) [51] or PCs (PCα1KO) [63] exist. While α^6^cre-Cacna1a KOs show impaired motor learning and memory consolidation but no motor impairment or SWDs, PCα1KOs develop a severe motor phenotype but occurrence of SWDs has not been reported. It was reported that the α^6^cre-Cacna1a KOs only have a scattered cre-expression in 75–86% of the cerebellar GCs [51] whereas quirky mice show an expression in more than 95% of cerebellar GCs (Supplementary Fig. 2). This difference in cre-expression could be responsible for the different phenotypes indicating that the small number of GCs expressing the P/Q-type calcium channel in α^6^cre-Cacna1a KO may compensate for most of the ataxic and epileptic phenotype seen in quirky mice. The differences found in the four mouse models based on the conditional knockout of the P/Q-type calcium channel show that similar genetic modifications in the cerebellar cortex can lead to diverse phenotypes and careful analysis is necessary to decipher the underlying network mechanisms.

**Fig. 6:**
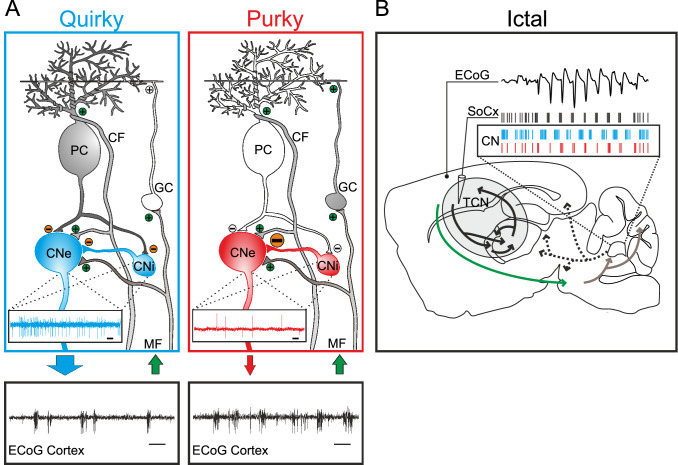
Schematic description of the cerebellar impact on SWDs in quirky and purky mice. **A** Excitatory cerebellar nuclei neurons (CNe) receive inhibitory inputs from Purkinje cells (PCs), but also from local inhibitory cerebellar nuclei neurons (CNi). Excitatory inputs via mossy fibers (MFs) or climbing fibers (CFs) originate from various precerebellar nuclei that convey information from the cortex and other structures (green arrow) to the cerebellum. White cells indicate the conditional knockout of the P/Q-type channel which renders their signal transmission. The large arrow exiting the CNe in quirky indicates a stronger excitatory drive on extracerebellar structures than in purky. This low excitatory drive in purky results in an increased number of SWDs in comparison to quirky mice (scale bar 10 s). We hypothesized that a loss of PC inhibition of CNi is responsible for an increased local inhibition of CNe and thereby the decreased firing rate in purky (scale bar 50 ms). Granule cell (GC). **B** Ictal events are characterized by SWDs in the ECoG which originate in the thalamo-cortical network (TCN, light grey oval). During this oscillatory activity, deep layer neurons of the primary somatosensory cortex (SoCx) fire action potentials rhythmically before the spike of the SWD. Cortical information is transmitted to precerebellar nuclei (green arrow) and from there to the cerebellum (grey arrows). These inputs are processed in the cerebellum and result in different rhythmic firing patterns in quirky and purky CN neurons (see box). Note, the example ECoG depicts a SWD in quirky mice. Example spikes for SoCx are based on [14]. For quirky (cyan) and purky (red) CN action potential firing was exemplified based on our results. CN neurons of purky mice fire rhythmically during or before the spike of the SWD like SoCx neurons while CN neurons in quirky mice fire rhythmically during the wave

We found that seizure-modulated neurons in the CN of quirky and purky mice fire rhythmically during SWDs as previously described for tottering mice or WAG/Rij and F344 rats [19, 52]. More specifically, we found that CN neurons fire only rhythmically during the wave of the SWD in quirky mice, whereas in purky CN neurons fire rhythmically during the spike of the SWD. Interestingly, phase-locking during the wave was described for tottering mice [19], while both forms of phase-locking were found in WAG/Rij and F344 rats [52]. A possible explanation for different firing patterns in quirky and purky mice could be that the external input to the CN is driving this firing pattern rather than the intrinsic activity of the CN (Fig. 6). External input enters the cerebellum either via mossy fibers from the brain stem and spinal cord or from climbing fibers ascending from the inferior olive [64, 65]. These excitatory pathways innervate PCs directly (climbing fibers) or indirectly (mossy fibers) but also bifurcate and directly innervate the CN. During the spike of the SWD TCN neurons fire action potentials phase-locked and this information is transmitted to the cerebellum (Fig. 6). In quirky mice, the PCs receive the rhythmic external information which is processed by PCs and transmitted to the CN resulting in rhythmic silencing of the CN by the purely GABAergic PCs. However, in purky mice, the processing power of PCs is missing due to extensive PC degeneration and hence the CN collaterals are directly conveying the cortical information to the CN leading to an unfiltered and rhythmic pattern of action potential firing as seen in the TCN. Noteworthy, it is also possible that a different population of CN neurons is responsible for the difference in rhythmic firing in quirky and purky mice. So far, it is not clear whether seizure-modulated neurons belong to a homologous population or whether different cell types receive similar external input that contribute to the rhythmic firing in quirky and purky mice.

Based on our electrophysiological data, we see that a reduction in CN activity could be a contributing factor for increased SWDs in an already epilepsy-prone TCN. When we chemogenetically decreased the activity of the CN, we found an increase in SWDs in quirky and purky mice. This is similar to a previous study where activation of the GABA_A_ receptor by muscimol increased SWDs in tottering [19]. Since the CN efferents to the TCN are mostly excitatory [66] the reduced output could shift the balance between excitation and inhibition inside the TCN towards inhibition which is believed to be critical for SWD generation [67–69]. Purky mice show an extensive loss of PCs [20] which are the sole output of the cerebellar cortex and an important source of inhibition to the CN [70]. One would assume that the resulting disinhibition in purky would increase the firing in CN neurons. Surprisingly, we found that purky mice showed a lower firing rate in CN neurons (Fig. 3). This agrees with another mouse model with an extensive loss of PCs called Purkinje cell degeneration (pcd) mice. Pcd mice show a reduction in CN firing due to a compensatory increase in parvalbumin positive cells [71]. This decrease in CN activity could be at least partially responsible for the more severe epileptic phenotype in purky mice (Fig. 6). It is noteworthy to mention that changes in CN and PC firing precision are believed to be responsible for ataxia in mouse models (i.e., Spinocerebellar ataxia type 6) that do not develop ASs [72]. There is no clear understanding of how movement disorders affect ASs or vice versa, although several mouse models that are used to study movement disorders often show ASs [73, 74] and these two seemingly unrelated disorders could be negatively affecting one another as seen in human patients [75]. Interestingly, approximately half of institutionalized patients with epilepsy also suffer from ataxia, a sign of prominent cerebellar dysfunction [76]. Furthermore, several studies in human patients found that patients with epilepsy also show cerebellar volume reduction, which could be either the predisposing factor for epilepsy or a result of recurrent seizures [77–79]. Interestingly, there are several reports of humans with lesions or tumors in the cerebellum that develop seizures and often experience recovery after resection [80, 81]. However, whether patients also have seizure-modulated cells in the cerebellar circuit is still unknown.

### The role of G protein-coupled receptors in absence seizures

GPCRs are one of the major interests in drug research due to the widespread expression in the brain and their implications for treatment of several disorders [82, 83]. Accordingly, the role of GPCRs has been tested in the treatment of SWDs in several studies targeting dopamine, opioid, glutamate, acetylcholine, noradrenaline, serotonin and endocannabinoid receptors [61, 84]. However, these studies only investigated the role of GPCR signaling in the TCN or after systemic injection but never considered regions outside the TCN. Here we show that the CN neurons are critically involved in the occurrence of SWDs and that pharmacological therapy using GPCR targeting drugs in the CN can have a reducing effect on SWD occurrence. Our data show that, although the TCN is responsible for the ictogenesis, other regions can be targeted for pharmacological therapy. We tested the anti-epileptic potential of CN activation via activation of G_q_-coupled DREADDs, mGluR1 and 5-HT2A pathway and found that general G_q_ and mGluR1 activation reduce SWD occurrence. Since we did not identify the specific cell population that is responsible for the antiepileptic effect, we cannot exclude that 5-HT2A activation had no effect due to receptor expression on a different cell type not involved in the antiepileptic effect. Nevertheless, these data provide evidence that increasing CN activity by activation of the native mGluR1 G receptor pathway can have antiepileptic potential. Interestingly, pharmacological blockage of inhibitory signaling in the CN has also been shown to decrease SWD occurrence, further highlighting that an increase in CN activity decreases SWDs [19].

### Closed-loop stimulation of CN neurons stops SWDs

We showed that within a detection delay of ~ 500 ms a single bilateral or unilateral 1 s blue light pulse was sufficient to abort SWDs within 200 ms with more than 90% efficiency (Fig. 6), in line with previously reported results in *tottering* and *C3H/HeOuJ* mice [19]. Stimulation of ChR2 was also used in the kainate mouse model to stop temporal lobe epilepsy by activation of the CN or cerebellar cortex while inhibition of the CN was ineffective [45, 46]. Although transient deactivation of the CN using DREADDs increased the occurrence of SWDs, we also found that brief photoinhibition by NpHR did not affect ongoing SWDs. Hence the contribution of CN firing is changing between preictal and ictal states since a decreased interictal CN output has a pro-epileptic effect. While lack of CN output does not affect maintenance of SWDs excitation can reliably disrupt the cortical oscillations (Fig. 6 and S4). Recent studies clearly show that CN stimulation is effective and can cease seizures not limited to a specific seizure type but has more possible use beyond SWDs. It is not clear how the CN activation terminates ongoing SWDs but one possible explanation could be that excitation of the CN in turn stimulates thalamic nuclei and thus disrupt the TCN rhythmicity seen during SWDs [14, 16]. Despite this direct interaction between CN and thalamus SWDs could not be stopped by optogenetic stimulation of cerebello-thalamic fibers [85]. More likely is that the stimulation of the CN results in a widespread desynchronizing stimulus due to the high connectivity of the CN.

While closed-loop stimulation opens a variety of chances to control seizures and especially pharmaco-resistant forms of seizures on-demand, the next step in treating patients is to predict and use stimulation paradigms to prevent generalization. While CN stimulation can be a target for directed preventive paradigms, there is no clear preictal pre-cursor that helps to identify SWDs prior to the beginning. Different approaches have been used to identify such a precursor in the EEG or ECoG, but these still need to be confirmed if they can predict SWDs reliably online [86–88]. Channelrhodopsin-2 as a tool has been extensively used and provided reliable results in this study, but it should be noted that there are several optogenetic probes beyond ChR2 that allow for control of GPCRs, yet with differential temporal features [89–91]. In future studies, these tools could help to understand GPCR modulation during SWDs and even allow a preventive and long-lasting seizure reduction due to slower kinetics than ChR2.

## Supplementary Information

Below is the link to the electronic supplementary material.

Supplementary file1 (PDF 920 KB)

## Data Availability

All materials and data supporting this study are available from the corresponding author (melanie.mark@rub.de) upon reasonable request.
