# Supplementary material for: Controlling absence seizures from the cerebellar nuclei via activation of the Gq signaling pathway
Source: Cell Mol Life Sci. 2022 Mar 19;79(4):197. doi: 10.1007/s00018-022-04221-5 (PMC8934336; doi:10.1007/s00018-022-04221-5)
Supplement: Supplementary file 1 — Supplementary file1 (PDF 920 KB) [file 18_2022_4221_MOESM1_ESM.pdf]

Supplemental materials for the manuscript

Controlling absence seizures from the cerebellar nuclei via activation of the G_q signaling pathway

Jan Claudius Schwitalla[1], Johanna Pakusch[1], Brix Mücher[2], Alexander Brückner[3], Dominic Depke[4], Thomas Fenzl[5], Chris I. De Zeeuw[6,7], Lieke Kros[6], Freek E. Hoebeek[8], Melanie D. Mark[1*]

[1]Department of Behavioral Neuroscience, Ruhr-University Bochum; 44801 Bochum, Germany.
[2]Department of Zoology and Neurobiology, Ruhr-University Bochum; 44801 Bochum, Germany.
[3]Institute of Physiology I, Medical Faculty, University of Bonn; 53127 Bonn, Germany.
[4]European Institute of Molecular Imaging, University of Münster; 48149 Münster, Germany.
[5]Department of Anesthesiology and Intensive Care, School of Medicine, Klinikum rechts der Isar, Technical University of Munich; 81675, Munich, Germany.
[6]Department of Neuroscience, Erasmus MC; 3015 AA Rotterdam, The Netherlands.
[7]Netherlands Institute for Neuroscience, Royal Dutch Academy for Arts and Sciences, 1105, BA Amsterdam, The Netherlands
[8]Department for Developmental Origins of Disease, Wilhelmina Children's Hospital and Brain Center, University Medical Center Utrecht; 3584 EA Utrecht, The Netherlands.

*Corresponding author: Melanie D. Mark, Department of Behavioral Neuroscience, Ruhr-University Bochum; 44801 Bochum, Germany. Email: melanie.mark@rub.de

Supplementary table 1: Statistical analysis corresponding to figure 1 and S1

Data	Groups	n	p-value	t or F-value	Statistical test
Peak frequency ictal	Quirky	5	0.016		two-tailed Mann-Whitney
	Purky	5			*U* test
Peak frequency interictal	Quirky	5	0.016		two-tailed Mann-Whitney
	Purky	5			*U* test
Sex difference	Mouse line		0.003	$F(1,24) = 10.675$	Two-way ANOVA with all pairwise multiple
	Sex		0.805	$F(1,24) = 0.0622$	comparisons (Bonferroni)
Quirky	male	7			
	female	9			
Purky	male	6			
	female	6			
Quirky Valproic acid	Drug		0.476	$F(1,9) = 0.522$	Two-way repeated measures ANOVA
	Time		0.385	$F(1,9) = 0.835$	
200 mg/kg		7			
Vehicle		4			
Quirky Ethosuximide	Drug		0.051	$F(2,19) = 3.498$	Two-way repeated measures ANOVA
	Time		< 0.001	$F(1,19) = 22.688$	with all pairwise multiple comparisons (Bonferroni)
50 mg/kg	pre	6	< 0.001	4.528	
	post	6			
100 mg/kg	pre	8	0.002	3.683	
	post	8			
Vehicle	pre	8	0.832	0.215	
	post	8			
Vehicle	post	8	0.005	3.415	
50 mg/kg	post	6			
Vehicle	post	8	0.004	3.562	
100 mg/kg	post	8			
Purky Valproic acid	Drug		0.325	$F(1,10) = 1.071$	Two-way repeated measures ANOVA
	Time		0.213	$F(1,10) = 1.771$	
200 mg/kg		6			
Vehicle		6			
Purky Ethosuximide	Drug		0.289	$F(3,22) = 1.332$	Two-way repeated measures ANOVA
	Time		< 0.001	$F(1,22) = 26.395$	with all pairwise multiple comparisons (Bonferroni)
50 mg/kg	pre	6	0.146	1.508	
	post	6			
100 mg/kg	pre	7	< 0.001	4.179	
	post	7			
200 mg/kg	pre	6	< 0.001	4.097	
	post	6			
Vehicle	pre	7	0.648	0.462	
	post	7			
Vehicle	post	7	0.065	2.726	

100 mg/kg	post	7			
Diurnal pattern of SWDs	Mouse line		0.043	*F*(1,5) = 7.310	Two-way repeated measures ANOVA
	Time		0.909	*F*(1,5) = 0.0144	
Quirky		4			
Purky		3			

Supplementary table 2: Statistical analysis corresponding to figure 2

Data	Groups	n	p-value	Statistical test
CN frequency	Control	53	0.0475	Kruskal-Wallis test with all pairwise multiple comparisons (Dunn's test)
	Quirky	31		
	Purky	23		
	Control	53	0.7585	
	Quirky	31		
	Control	53	0.0402	
	Purky	23		
	Quirky	31	0.3417	
	Purky	23		
CN CV	Control	53	< 0.001	Kruskal-Wallis test with all pairwise multiple comparisons (Dunn's test)
	Quirky	31		
	Purky	23		
	Control	53	< 0.001	
	Quirky	31		
	Control	53	0.0026	
	Purky	23		
	Quirky	31	0.0182	
	Purky	23		
CN mean CV2	Control	53	0.0055	Kruskal-Wallis test with all pairwise multiple comparisons (Dunn's test)
	Quirky	31		
	Purky	23		
	Control	53	0.0186	
	Quirky	31		
	Control	53	0.0339	
	Purky	23		
	Quirky	31	0.9999	
	Purky	23		
CN burst index	Control	53	0.00895	Kruskal-Wallis test with all pairwise multiple comparisons (Dunn's test)
	Quirky	31		
	Purky	23		
	Control	53	0.0092	
	Quirky	31		
	Control	53	0.9946	
	Purky	23		
	Quirky	31	0.0749	
	Purky	23		

Supplementary table 3: Statistical analysis corresponding to figure 3 and S3

Data	Groups	n	p-value	t or F-value	Statistical test
Quirky DREADD G_q	Mouse line		0.317	*F*(1,10) = 1.108	Two-way repeated measures ANOVA
	Time		0.541		

				$F(1,10)$ = 0.401	with all pairwise multiple comparisons (Bonferroni)
Vehicle	pre	6	0.258	1.198	
	post	6			
CNO	pre	6	0.063	2.094	
	post	6			
Vehicle	post	6	0.032	2.307	
CNO	post	6			
Vehicle	pre	6	0.493	0.699	
CNO	pre	6			
Quirky DREADD $G_{i/o}$	Mouse line		0.087	$F(1,12)$ = 3.466	Two-way repeated measures ANOVA
	Time		0.004	$F(1,12)$ = 13.053	with all pairwise multiple comparisons (Bonferroni)
Vehicle	pre	7	0.825	0.226	
	post	7			
CNO	pre	7	< 0.001	5.336	
	post	7			
Vehicle	post	7	0.002	3.58	
CNO	post	7			
Vehicle	pre	7	0.726	0.356	
CNO	pre	7			
Purky DREADD G_q	Mouse line		0.022	$F(1,10)$ = 7.331	Two-way repeated measures ANOVA
	Time		0.482	$F(1,10)$ = 0.534	with all pairwise multiple comparisons (Bonferroni)
Vehicle	pre	6	0.311	1.068	
	post	6			
CNO	pre	6	0.062	2.102	
	post	6			
Vehicle	post	6	0.002	3.514	
CNO	post	6			
Vehicle	pre	6	0.472	0.733	
CNO	pre	6			
Purky DREADD $G_{i/o}$	Mouse line		0.091	$F(1,10)$ = 3.492	Two-way repeated measures ANOVA
	Time		0.011	$F(1,10)$ = 9.626	with all pairwise multiple comparisons (Bonferroni)
Vehicle	pre	6	0.666	0.445	
	post	6			
CNO	pre	6	0.003	3.942	
	post	6			
Vehicle	post	6	0.01	2.90	
CNO	post	6			
Vehicle	pre	6	0.798	0.260	
CNO	pre	6			
Quirky	Pre	6	< 0.001	F(2,17) = 44.125	One-way repeated measures ANOVA with all pairwise multiple comparisons (Bonferroni)
	CNO	6			
	ETX	6			
	Pre	6	< 0.001	6.341	
	CNO	6			
	Pre	6	0.053	2.832	
	ETX	6			
	CNO	6	< 0.001	9.173	

	ETX	6			
Purky	Pre<br>CNO<br>ETX	6<br>6<br>6	0.03	F(2,17) = 5.076	One-way repeated measures ANOVA with all pairwise multiple comparisons (Bonferroni)
	Pre<br>CNO	6<br>6	0.099	2.475	
	Pre<br>ETX	6<br>6	1.0	0.501	
	CNO<br>ETX	6<br>6	0.042	2.976	
Quirky mCh	Mouse line		0.446	F(1,8) = 0.642	Two-way repeated measures ANOVA
	Time		0.720	F(1,8) = 0.138	
Vehicle		5			
CNO		5			
Purky mCh	Mouse line		0.340	F(1,6) = 1.074	Two-way repeated measures ANOVA
	Time		0.427	F(1,6) = 0.725	
Vehicle		4			
CNO		4			

Supplementary table 4: Statistical analysis corresponding to figure 4

Data	Groups	n	p-value	t or F-value	Statistical test
Quirky DHPG	Drug		0.448	*F*(1,9) = 0.628	Two-way repeated measures ANOVA with all pairwise multiple comparisons (Bonferroni)
	Time		0.011	*F*(1,9) = 10.278	
Vehicle	pre<br>post	5<br>5	0.950	0.0645	
DHPG	pre<br>post	6<br>6	< 0.001	4.826	
DHPG<br>Vehicle	post<br>post	5<br>6	0.024	2.501	
DHPG<br>Vehicle	pre<br>pre	5<br>6	0.253	1.187	
Purky DHPG	Drug		0.811	*F*(1,14) = 0.0592	Two-way repeated measures ANOVA with all pairwise multiple comparisons (Bonferroni)
	Time		< 0.001	*F*(1,14) = 33.489	
Purky Vehicle	pre<br>post	8<br>8	0.547	0.617	
Purky DHPG	pre<br>post	8<br>8	< 0.001	8.801	
DHPG<br>Vehicle	post<br>post	8<br>8	0.056	2.062	
DHPG<br>Vehicle	pre<br>pre	8<br>8	0.130	1.594	
Quirky TCB-2	Drug		0.787	*F*(1,10) = 0.0767	Two-way repeated measures ANOVA
	Time		0.732	*F*(1,10) = 0.124	

Quirky Vehicle		6			
Quirky TCB-2		6			
Purky TCB-2	Drug		0.848	*F*(1,10) = 0.0385	Two-way repeated measures ANOVA
	Time		0.056	*F*(1,10) = 4.670	
Purky Vehicle		7			
Purky TCB-2		5			
Freely moving vs Head-fixed	Mouse line		0.003	*F*(1,71) = 4.670	Two-way ANOVA with all pairwise multiple comparisons (Bonferroni)
	Condition		<0.001	*F*(1,71) = 40.889	
Quirky	Freely moving	16			
	Head-fixed	26			
Purky	Freely moving	12			
	Head-fixed	21			

Supplementary table 5: Statistical analysis corresponding to figure 5, S4 to S6

Data	Groups	n	p-value	t or F-value	Statistical test
Quirky ChR2			< 0.001	F(3,14) = 199.763	One-way ANOVA with all pairwise multiple comparisons (Bonferroni)
	Bilateral	6	< 0.001	17.196	
	mCh	3			
	Unilateral	3	< 0.001	15.583	
	mCh	3			
	Bilateral	6	< 0.001	18.867	
	Sham	6			
	Unilateral	3	< 0.001	16.203	
	Sham	6			
	Bilateral	6	1.0	0.798	
	Unilateral	3			
	Sham	3	0.570	1.791	
	mCh	3			
Purky ChR2			< 0.001	F(3,11) = 268.349	One-way ANOVA with all pairwise multiple comparisons (Bonferroni)
	Bilateral	5	< 0.001	19.900	
	mCh	3			
	Unilateral	3	< 0.001	18.703	
	mCh	3			
	Bilateral	5	< 0.001	21.312	
	Sham	4			
	Unilateral	3	< 0.001	19.685	
	Sham	4			
	Bilateral	5	1.011	1.0	
	Unilateral	3			
	Sham	4	0.309	1.0	
	mCh	3			
Quirky NpHR	Group		0.939	*F*(1,3) = 0.00679	Two-way repeated measures ANOVA

	Time		0.171	*F*(1,3) = 3.218	
	NpHR	4			
	Sham	4			
Purky NpHR	Group		0.347	*F*(1,3) = 1.237	Two-way repeated measures ANOVA
	Time		0.658	*F*(1,3) = 0.477	
	NpHR	4			
	Sham	4			
Quirky ChR2 Short stimulation			< 0.001	F(4,8) = 27.367	One-way repeated measures ANOVA with all pairwise multiple comparisons (Bonferroni)
	100 ms	3	< 0.001	8.609	
	Orange	3			
	50 ms	3	< 0.001	7.310	
	Orange	3			
	30 ms	3	< 0.001	7.808	
	Orange	3			
	10 ms	3	0.175	2.982	
	Orange	3			

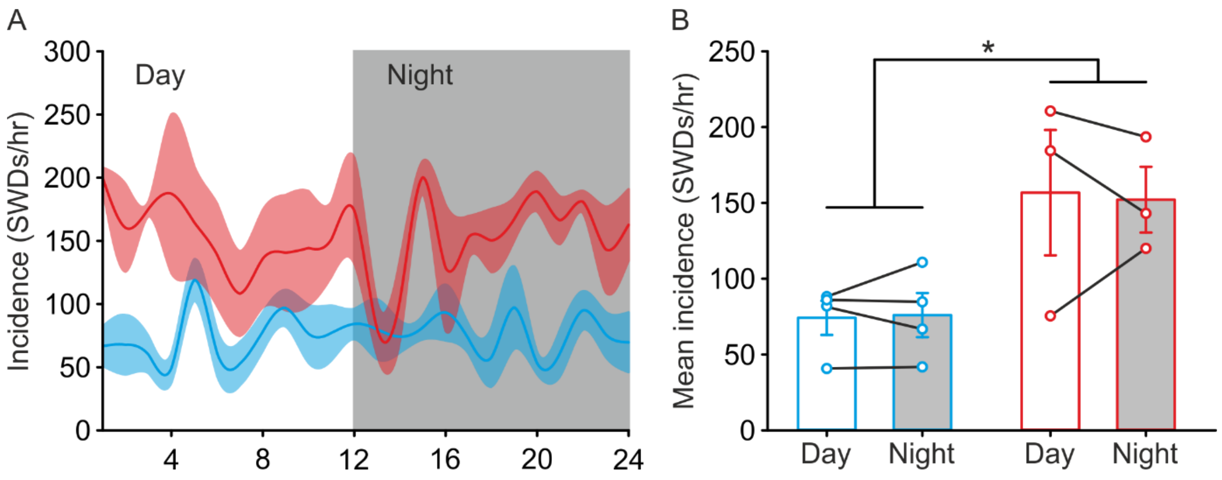

Supplementary Fig. 1 SWDs do not show a diurnal cycle in quirky and purky mice (related to Figure 1)

(**A**) Number of seizures during the day (white) and night (grey) in quirky (cyan; n = 4) and purky (red; n = 3) mice. Thick lines represent incidence per hour and shadowed areas represent ± SEM. **(B)** Quirky and purky mice showed no significant difference in the mean number of SWDs between day and night (two-way repeated measures ANOVA). Bars represent mean ± SEM and individual animals are represented as circles. For detailed statistical analysis see Supplementary Table 1.

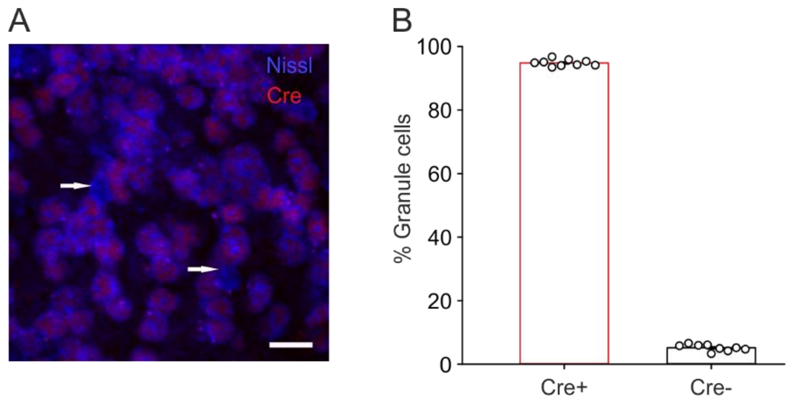

Supplementary Fig. 2 Expression of Cre recombinase in cerebellar granule cells in quirky mice (related to Figure 1)

(A) Parasagittal section of the granule cell layer in quirky mice stained for Nissl (blue) and Cre recombinase (red). White arrows depict Cre negative cells. Scale bar 10 µm. **(B)** Quirky mice show approximately 5% Cre negative cells (3 areas in n = 3 animals). Bars represent mean ± SEM and areas are represented as circles.

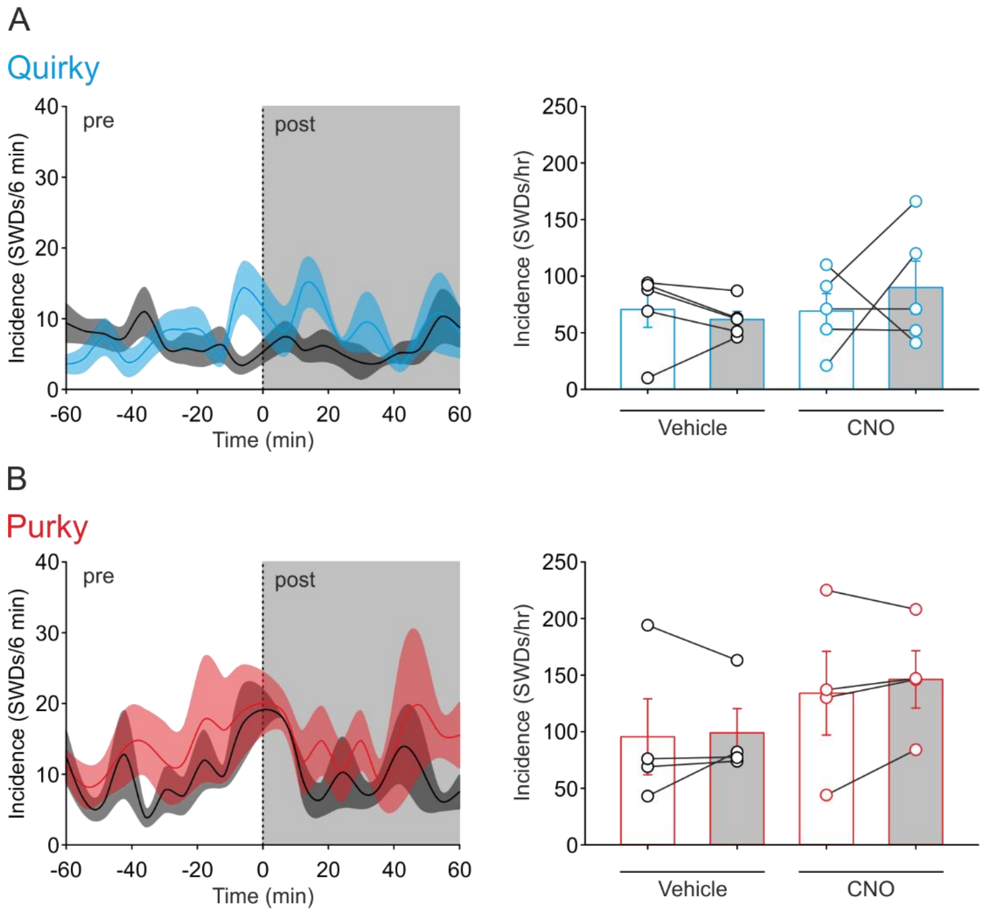

Supplementary Fig. 3 SWDs are not affected by CNO injection (related to Figure 3)

(A and B) Change in the number of SWDs after (post, grey box) intraperitoneal injection of vehicle (black) and 1 mg/kg clozapine N-oxide (CNO) in (A) quirky (cyan, n = 4) and (B) purky (red, n = 4)

mice expressing mCherry (mCh) in the CN. Thick lines represent mean incidence and shadowed areas represent ± SEM. Number of SWDs before (white bar) and after vehicle or CNO injection (grey bar) in quirky and purky mice are not significantly changed (two-way repeated measures ANOVA). Bars represent mean ± SEM and individual animals are represented as circles. For detailed statistical analysis see Supplementary Table 3.

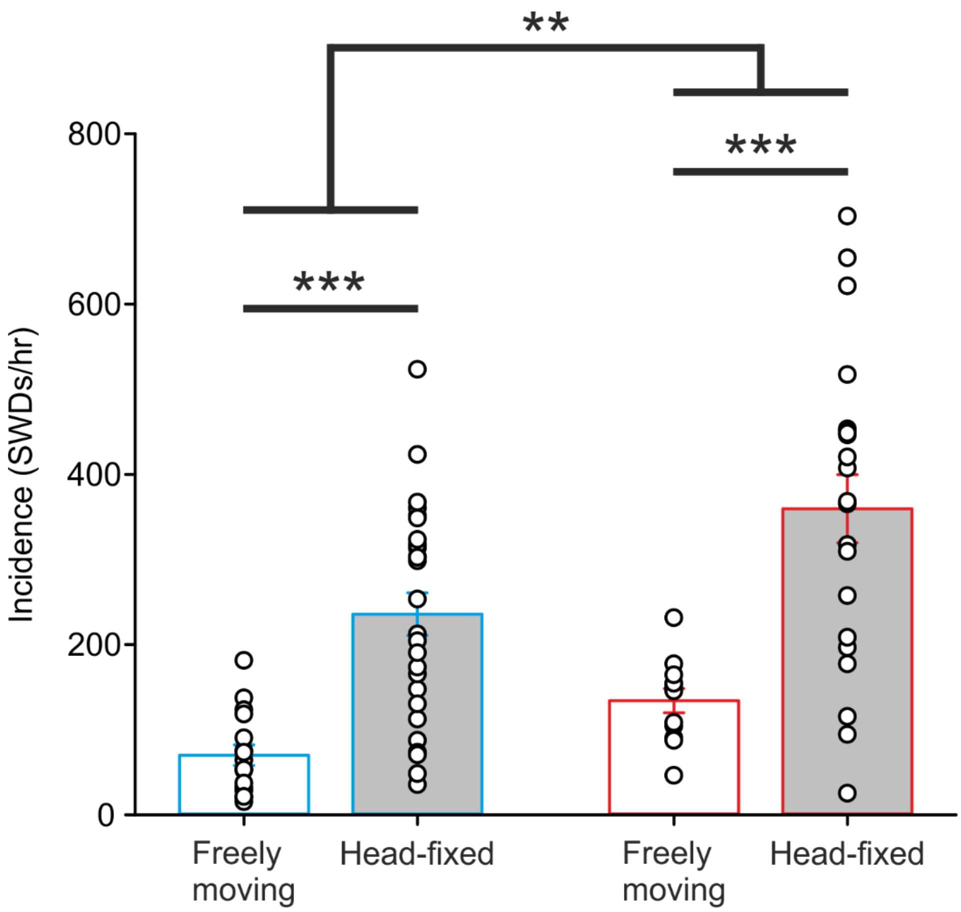

Supplementary Fig. 4 Comparison of SWDs in freely moving and head-fixed quirky and purky mice

One-hour ECoG recordings from freely moving quirky (n=16) and purky (n=12) mice and head-fixed quirky (n=26) and purky (n=21) mice. Head-fixed mice show significantly more SWDs per hour in comparison to freely-moving animals (two-way ANOVA with pairwise Bonferroni test). The typical significantly higher number of SWDs in purky can still be detected in head-fixed animals. Recordings are pre recordings from uninjected animals that were used in part for this manuscript. Bars represent mean ± SEM and individual animals are represented as circles. For detailed statistical analysis see Supplementary Table 4.

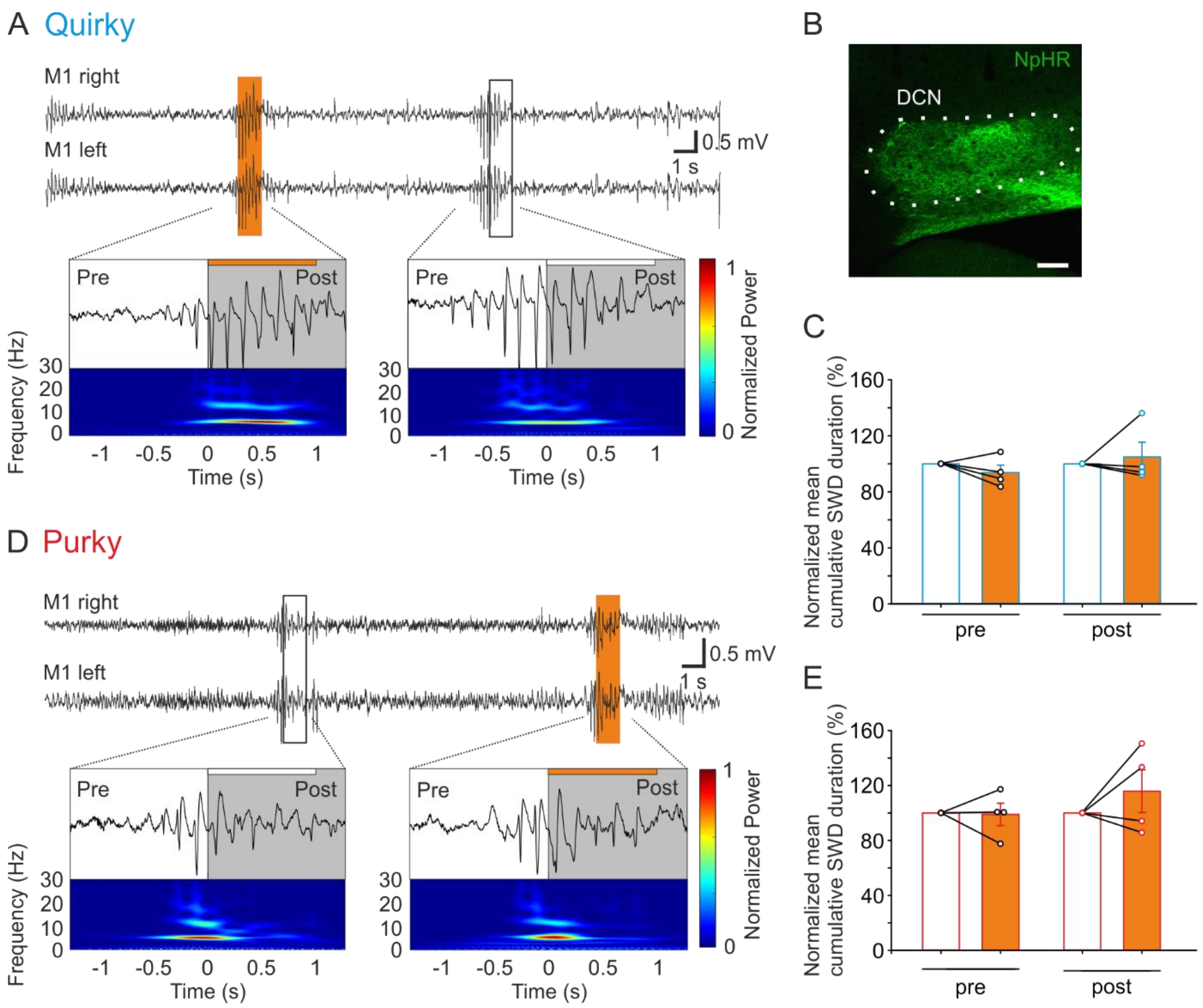

Supplementary Fig. 5 Seizure duration is unaltered after CN neuron inhibition in quirky and purky mice (related to Figure 5)

(A and D) Representative example of electrocorticogram activity (M1 left bottom trace and M1 right top trace) and power spectrum analysis before (pre, white rectangle) and after (grey rectangle) 1 s sham (no stimulation, small white box) or orange light (620 nm) halorhodopsin (NpHR) stimulation (small orange box) of CN neurons during ongoing SWDs in (A) quirky (cyan) and (D) purky (red) mice. **(B)** Coronal section depicting representative expression of NpHR inside the CN of a quirky mouse. Scale bar 100 µm. **(C and E)** Mean cumulative SWD duration before stimulation (pre) and after orange (620 nm) light NpHR stimulation (post) for sham (white bar) and halorhodopsin (NpHR, orange bar) normalized to sham stimulation pre and post in (C) quirky (NpHR n=460 and Sham n = 529 from 4 animals) and (E) purky (NpHR n = 334 and Sham n = 307 from 4 animals) mice are not significantly changed (two-way repeated measures ANOVA). Bars represent mean ± SEM and individual animals are represented as circles. For detailed statistical analysis see Supplementary Table 5.

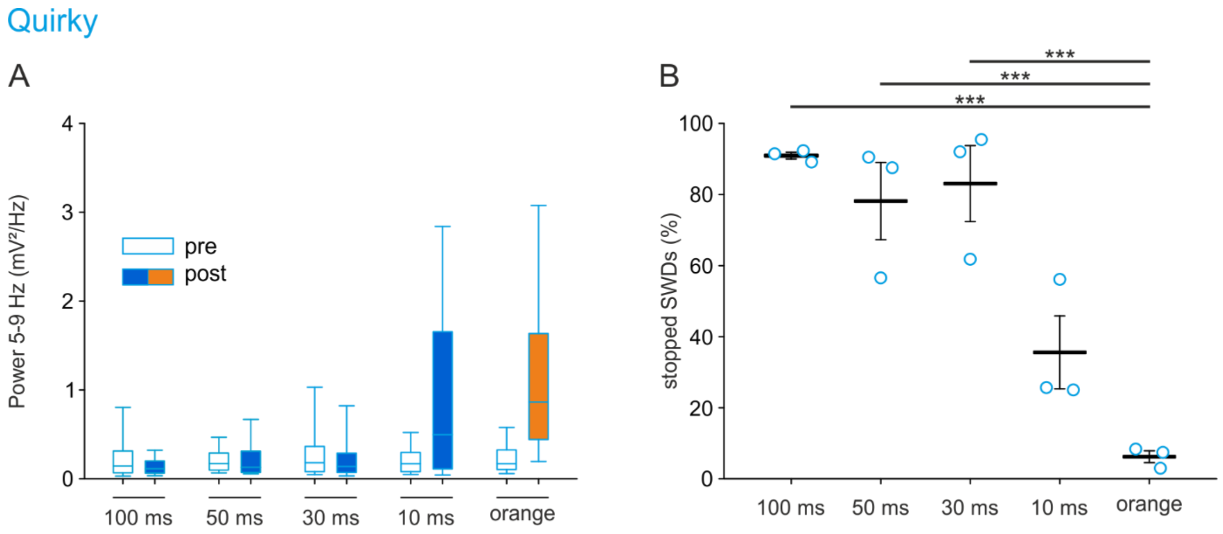

Supplementary Fig. 6 Brief excitation of the CN neurons can stop seizures (related to Figure 5) **(A)** Boxplot represent seizure specific power pre (white boxplot) and after (blue and orange boxplot) 100 ms (n = 107 from 3 animals), 50 ms (n = 83 from 3 animals), 30 ms (n = 115 from 3 animals), 10 ms (n = 132 from 3 animals) blue (465 nm) and orange (620 nm, n = 97 from 3 animals) light stimulation of CN neurons expressing channelrhodopsin-2 during ongoing SWDs in quirky mice. Orange (100 ms) was used as a control since channelrhodopsin2 (ChR2) is not sensitive to orange light. Seizure specific power was decreased after 100 ms, 50 ms and 30 ms blue light ChR2 stimulation. **(B)** Percentage of successfully stopped seizures was significantly higher after 100 ms, 50 ms, 30 ms than orange (100 ms) light stimulation (n = 3 for all tested durations and orange light, one-way repeated measures ANOVA with all pairwise Bonferroni test). Lines represent mean ± SEM and individual animals are represented as circles. For detailed statistical analysis see Supplementary Table 5. *** *P*-value < 0.001.
